# Catalytic performance of the Ce-doped LaCoO_3_ perovskite nanoparticles

**DOI:** 10.1038/s41598-020-71869-z

**Published:** 2020-09-14

**Authors:** Anees A. Ansari, Syed F. Adil, Manawwer Alam, N. Ahmad, Mohamed E. Assal, Joselito P. Labis, Abdulrahman Alwarthan

**Affiliations:** 1 King Abdullah Institute for Nanotechnology, King Saud University, Riyadh, 11451 Saudi Arabia; 2 Department of Chemistry, King Saud University, Riyadh, 11451 Saudi Arabia

**Keywords:** Materials for energy and catalysis, Chemistry, Materials science, Nanoscience and technology

## Abstract

A series of La_1-x_Ce_x_CoO_3_ perovskite nanoparticles with rhombohedral phases was synthesized via sol–gel chemical process. X-ray diffraction (XRD), Transmission Electron Microscopy (TEM), Electron Diffraction Spectroscopy (EDS), Thermogravimetric Analysis (TGA), UV–Vis spectroscopy, Fourier Transform Infrared spectra (FTIR), Nitrogen Adsorption/desorption Isotherm, Temperature Program Reduction/Oxidation (TPR/TPO), X-ray Photoelectron Spectroscopy (XPS) techniques were utilized to examine the phase purity and chemical composition of the materials. An appropriate doping quantity of Ce ion in the LaCoO_3_ matrix have reduced the bond angle, thus distorting the geometrical structure and creating oxygen vacancies, which thus provides fast electron transportation. The reducibility character and surface adsorbed oxygen vacancies of the perovskites were further improved, as revealed by H_2_-TPR, O_2_-TPD and XPS studies. Furthermore, the oxidation of benzyl alcohol was investigated using the prepared perovskites to examine the effect of ceria doping on the catalytic performance of the material. The reaction was carried out with ultra-pure molecular oxygen as oxidant at atmospheric pressure in liquid medium and the kinetics of the reaction was investigated, with a focus on the conversion and selectivity towards benzaldehyde. Under optimum reaction conditions, the 5% Ce doped LaCoO_3_ catalyst exhibited enhanced catalytic activity (i.e., > 35%) and selectivity of > 99%, as compared to the other prepared catalysts. Remarkably, the activity of catalyst has been found to be stable after four recycles.

## Introduction

With the advancements of science and nanotechnology, researchers have been continuously trying to establish different techniques for benzaldehyde production^1^. Benzaldehyde production is of vital importance from the scientific and industrial point of views. It is one of the most valuable aromatic aldehydes and versatile intermediates used in various chemical industries, such as pharmaceuticals, perfumery, dyestuff, and agrochemical industries. Among the various aspects in the production of benzaldehyde, three features are of utmost importance: (i) environmentally clean and green oxidants, (ii) non-toxic solvents, and (iii) low cost catalysts with high activity and selectivity^2,3^. Previously, the catalyst used for benzaldehyde production/generation were inorganic and organic oxidants, such as chromium trioxide, ammonium permanganate, and tert-butyl hydroperoxide. However, these chemicals are inherently toxic, expensive, corrosive, thus resulting to many environmental issues and concerns^4^. In view of this, enormous efforts have been devoted to develop a more environmentally friendly catalytic systems to decrease the drawbacks of the usual traditional oxidation approaches^5^. Therefore, a much needed alternative shift must be geared towards the use of clean oxidants, such as aqueous H_2_O_2_ and molecular O_2_, which has attracted a considerable amount of attention, because of its economic and environmental advantages^6^. Molecular oxygen is the most desirable oxidant because it is cheap, safe, readily available, and produces water as the sole byproduct^7^. Conventionally, many types of organic solvents, such as benzene, chloroform, toluene, acetonitrile, acetone, and xylene are used in benzaldehyde production via in alcohol oxidation^8^.

In an ideal oxidation process for benzaldehyde production, the catalyst should be eco-friendly, can easily be prepared, and has long term stability. In addition, easy product separation, recycling, and high conversion, besides selectivity, are some of its important parameters. Among catalytic oxidation reactions, heterogeneous catalysis has been widely studied, because of its long term stability and excellent performance. Selective oxidation of alcohols to carbonyl compounds over heterogeneous catalysts using molecular oxygen (aerobic oxidation) has attracted significant attentions from the viewpoint of green and sustainable chemical processes^9–11^. Many studies have been focused on the aerobic oxidation of alcohols using noble metal^12,13^ and transition metal catalysts^14,15^ with or without supports^6,16^. This is because they show high intrinsic advantages, in terms of effective catalytic ability, product selectivity, easy recovery and reusability. Alternatively, there is still growing consensus among researchers to explore the use of less expensive transition metals, composite oxides, metal oxides, and mixed metal oxides of transitional metals, such as Mn, Ni, Cu and Zn containing catalysts, although their catalytic activities are reportedly poorer than that of the noble metals. In fact, less expensive metals offer a cost effective alternative to the noble metal catalysts, and that their low catalytic activity may provide more insights into a reaction mechanism^17,18^.

Among the metal oxide based catalysts, ABO_3_ perovskite materials have unique physicochemical properties, such as good chemical/thermal stability, unique magnetic property, high ionic conductivity and excellent catalytic performance^19,20^. In comparing to bare metal oxides, perovskite-type oxides are associated with transition metal ions, which have variable oxidation states in their crystal structures. These variable oxidation states of transition metals assist in redox reaction mechanism. Generally, ceria has been exhibiting variable oxidation states. This is the unusual property of the ceria, which then creates non-stoichiometric defects sites resulting to high oxygen species transportation. High mobility of oxygen species reduces when the perovskites is at lower temperature. Further, perovskites at nanoscale along with hollow structure demonstrates unusual physiochemical properties, that are different from their bulk counterparts^21^.

In this study, we report the process of synthesizing Ce-doped LaCoO_3_ nanoparticles and their physiochemical properties at room temperature. In the present work, we introduced Ce ions into the LaCoO_3_ perovskite structure, due to high oxygen storage capacity and oxygen conversion ability of ceria at lower temperature. This direction is in line with the work of other researchers, who were reporting that doping increases the surface area, decreases grain sizes, and improves their optical and oxidation/reduction characteristics of the counterpart metal oxides^22,23^. Doping in semiconductor metal oxides is a powerful method to tailor the crystallographic, optoelectronic, magnetic, and redox properties, which then facilitates the fabrication of many optoelectronic devices^24–31^. It is interesting, therefore, to inspect the impact of Ce ion-doping on LaCoO_3_ symmetry, its distortion in the crystal structure, grain size and redox characteristics of the as-prepared perovskites. This study is extremely vital to the understanding of the effects of these dopant on the structural and texture properties of this type of perovskites. Here, we systematically presented the crystallographic, thermos-chemical, optical and redox (temperature program reduction/oxidation) properties of Ce-doped LaCoO_3_ nanoparticles to understand the role of Ce doping on physiochemical properties, which are responsible in increasing the catalytic performances of the perovskite materials. The Ce-doped LaCoO_3_ perovskites were prepared by co-precipitation method, and were comprehensively characterized using XRD, Field Emission-TEM, EDX, TGA, FTIR, UV/Vis absorption, and redox behavior based on H_2_-consumption through temperature program reduction technique.

## Experimental detail

### Synthesis of catalysts

Specifically, La_1-x_Ce_x_CoO_3_ (x = 0.0, 0.05, 0.07, and 0.1) nanoparticles were synthesized by a “citrate based” co-precipitation method. In this typical reaction, an equal volume (1:1 molar ratio) of La(NO_3_)_3_·7H_2_O (99.99%, BDH Chemicals Ltd, England) and Co(NO_3_)_2_·6H_2_O (99.9% E-Merck, Germany) were mixed together in 50 mL Milli Q water solution and kept under magnetic stirring on a hot plate at 80 °C to achieve a homogenous mixture. A hot solution of cerium nitrate was slowly added into the solution mixture. In another solution, 10 wt% excess aqueous solution of citric acid monohydrate (98.5%, E-Merck, Germany) was injected into the solution for complexation. After this, the solution mixture was transferred into a 250 mL round bottle flask fitted with reflux condenser for complete complexation up to 5–6 h. A quantity of NH_4_OH (99.99%, BDH Chemicals Ltd, England) was introduced to hydrolyze the solution for precipitation reaction. Later on, the obtained brown black colored precipitates were separated by centrifugation, washed many times with water to remove excess quantity of ammonium and nitrate ions, and finally dried at 250 °C, and finally calcined in air at 800 °C for 6 h to obtain the perovskites. This procedure was followed for synthesis of various concentrations of Ce-doped La_1-x_Ce_x_CoO_3_ perovskites.

### Characterizations

The powder samples were characterized XRD with the use of Rigaku D 2,500 diffractometer using the Cu Kα radiation (λ = 0.154 nm, 40 kV, 40 mA). The FTIR spectra were recorded on the Perkin-Elmer 580B IR spectrometer using the KBr pellet technique. UV/Vis spectra were recorded from the Perkin-Elmer Lambda-40 Spectrophotometer. The thermal decompositions of the perovskite precursors were performed simultaneously by thermogravimetric-thermal analysis (TG-DTG) using Mettler Toledo TGA/DSC 1 STAR^e^ thermal analyzer (Switzerland) between 50 and 900 °C at the heating rates of 20 °C min^−1^ in nitrogen atmosphere at a flow rate of 20 mL min^−1^. The morphology was checked using the Field Emission TEM, equipped with EDX (JEM-2100F, JEOL, Japan) operating at an accelerating voltage of 200 kV. The BET surface areas of the calcined catalysts were measured using the Micromeritics TriStar 3000 BET Analyzer, taking a value of 0.162 nm^2^ for the cross-sectional area of the N_2_ molecule adsorbed at 77 K. The sample degassing was carried out at 300 °C prior to measuring the adsorption isotherms.

The redox properties (H_2_-TPR and O_2_-TPO) were recorded using a chemisorption apparatus (Micromeritics AutoChem II 2920), equipped with a thermal conductivity detector. In this measurement, about 25 mg of sample was loaded in a U shaped quartz tube (6 mm ID). Samples were packed in the tube by quartz wool plugs and a thermocouple is inserted to measure the bed temperature. The samples were initially flushed in Argon at 300 °C for 60 min in order to eliminate the adsorbed water, and then cool to room temperature. H_2_-TPR was performed using a mixture of 10% H_2_/Ar at a flow rate of 20 mL min^−1^. The sample tube was heated at the ramping rate of 10 °C min^−1^ from 50 to 800 °C. After the reduction, sample was cooled to room temperature; and was then exposed to 10% O_2_/He for the oxidation (O_2_-TPO) at the same operating condition.

The XPS spectra were recorded using the PerkinElmer PHI 5000C system equipped with a hemispherical electron energy analyzer. Using the Mg K_*alpha*_ anode (h*v* = 1,253.6 eV), the XPS was operated at 15 kV and 20 mA. The binding energy (BE) scale was referenced to the C *1s* peak (~ 284.6 eV) arising from adventitious carbon in the sample.

### Catalytic reactions

The liquid-phase aerobic oxidation reaction of benzyl alcohol was carried out at atmospheric pressure in a magnetically stirred three-necked flask equipped with reflux condenser and thermometer. In brief, 200 mg catalyst, 2 mmol benzyl alcohol and 10 mL toluene (solvent) were mixed transferred in 50 mL flask. Prior to the oxidation process, the reaction mixture was purged in Argon gas for 1 h. The mixture was immersed in an oil bath and heated to 100 °C. The oxygen gas was bubbled at a flow rate of 20 mL min^−1^ into the pre-charged flask to start the reaction. The liquid products were collected every 2 h, separated by centrifuging, and analyzed by Agilent Gas Chromatograph 7890A, equipped with FID and HP-PONA capillary column. Using the equations below, the conversions and selectivities were calculated by the peak area, using Undecane as an internal standard. The recyclability of the highly active catalyst, La_0.95_Ce_0.05_CoO_3_, was also investigated^32^.

1 $${\text{Conversion }}\left( \% \right) \, = {\text{ No}}.{\text{ of moles reacted/No}}.{\text{ of moles remaining }} \times { 1}00$$

2 $${\text{Selectivity }}\left( \% \right) \, = {\text{ No}}.{\text{ of moles of desired product/No}}.{\text{ of moles remaining }} \times { 1}00$$

3 $${\text{Specific Activity }} = {\text{ mmoles of reactant }} \times {\text{ Conversion product/Weight of catalyst }}\left( {\text{g}} \right) \, \times {\text{ Reaction time }}\left( {\text{h}} \right)$$

4 $${\text{Turn Over Number }}\left( {{\text{TON}}} \right) = {\text{ No}}.{\text{ of moles of desired product formed/No}}.{\text{ of active sites}}$$

5 $${\text{Turn Over Frequency }}\left( {{\text{TOF}}} \right) \, = {\text{ Turn Over Number}}\left( {{\text{TON}}} \right){\text{/Reaction time}}$$

## Results and discussion

### Physiochemical properties of perovskite

**Figure 1:**
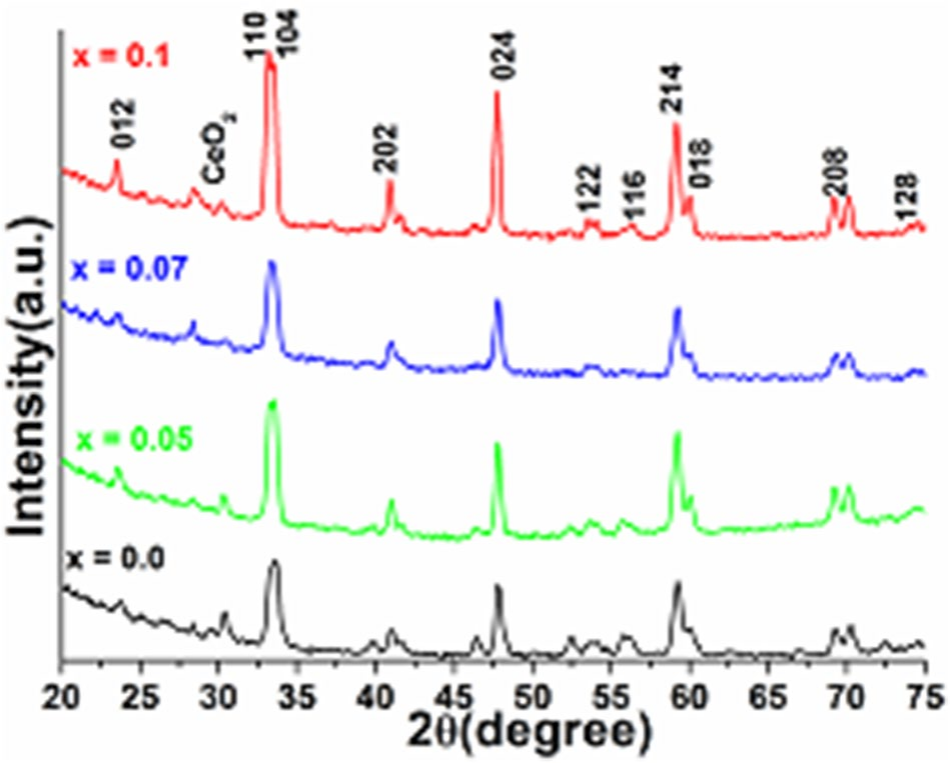
X-ray diffraction pattern of LaCoO_3_, La_0.95_Ce_0.05_CoO_3_, La_0.93_Ce_0.07_CoO_3_ and La_0.90_Ce_0.10_CoO_3_ nanoparticles.

The XRD patterns and calculated lattice parameters of the pre-calcined powder are presented in Fig. 1 and Table 1. All diffraction lines corresponding to (012), (110), (104), (202), (024), (122), (116), (214), (018), (208) and (128) planes are closely match to that of the rhombohedral phase of LaCoO_3_ perovskite (JCPDS Card No. 04-0848; 048-0123), and there are all in good agreement with the reported data^23,33^. The XRD peak broadening suggests that the as-prepared perovskites are well-crystalline and small in grain sizes (~ 100 nm), although, weak intensity of CeO_2_ peak is also observed for 7% and 10% Ceria dopings. Since CeO_2_ peak is not seen in the low concentration (5%) doping of ceria, it shows that low concentration can be accommodated in LaCoO_3_ framework^31,34^. On the addition of Ce^3+^ ions into the LaCoO_3_ structure, the reflection lines are slightly shifted towards higher 2θ angles, signifying decreases in the lattice parameters. Additionally, substitution of smaller radius Ce^3+^ (1.34 Å) ions at a site into LaCoO_3_ crystal distorts the crystal lattice due to a reduction in crystal spacing distance^35,36^. The observed discrepancy in lattice parameters may be due to ion-radius mismatch on the substitution of smaller radius ion at the La^3+^ site, which is in good agreement with the earlier reports (Table 1)^37,38^. The cell parameter “a”, for example, reduces with increasing Ce^3+^ content, as the ionic radius of the Ce^3+^ ion is smaller than that of the La^3+^ ion^30,39,40^. The structural alteration of the rhombohedral phase of the perovskite phase is a function of the size of replacing lanthanides, which generate the oxygen vacancies because of lattice distortion^41^. The cation replacement at the La^3+^ sites leads to different ion and vacancy ordering. This can result in deviations in the catalytic activities. It is observed that the activity of the as-prepared catalysts has improved after increasing the substitution concentration.

**Table 1:** Comparative analysis of lattice parameters of the perovskites.

Samples	a (Å)	c (Å)	C/k ratio
LaCoO_3_	5.527	13.384	2.421
La_0.95_Ce_0.05_CoO_3_	5.463	13.576	2.485
La_0.93_Ce_0.07_CoO_3_	5.444	13.617	2.498
La_0.90_Ce_0.10_CoO_3_	5.436	13.874	2.552

**Figure 2:**
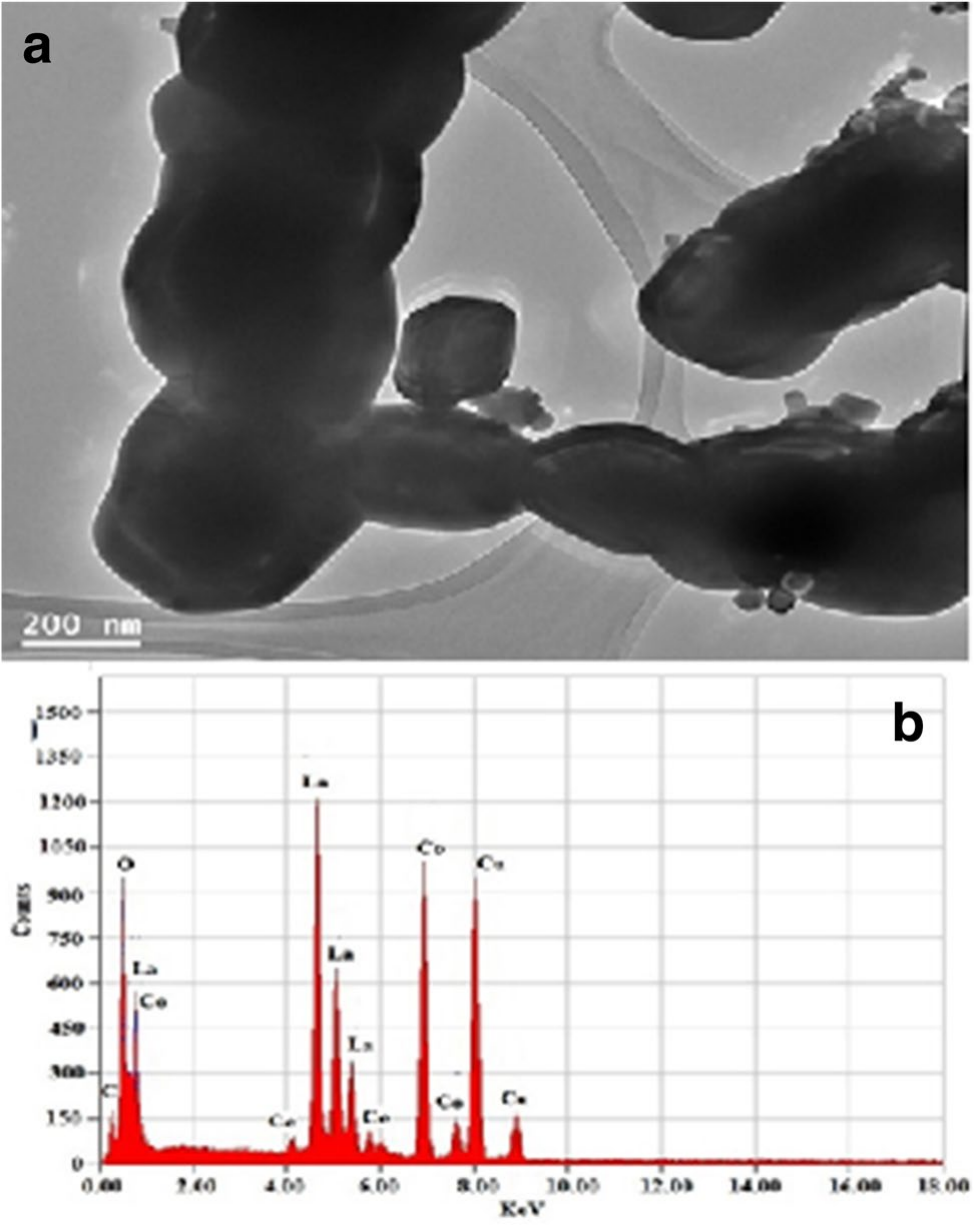
(**a**) TEM micrographs and (**b**) EDX spectrum of La_0.95_Ce_0.05_CoO_3_ nanoparticles.

The TEM–EDX of La_0.95_Ce_0.05_CoO_3_ nanoparticles is shown Fig. 2. As shown in Fig. 2a, the product is nanocrystal with 135–200 nm wide and shows rope-like structure in which Ce doped with uncontrolled size. Moreover, EDX spectrum of La_0.95_Ce_0.05_CoO_3_ clearly shows the peaks of the expected elements, such as La Co, Ce, and O throughout the whole structure, which is well-matched with the chemical composition of La_0.95_Ce_0.05_CoO_3_ nanoparticles (Fig. 2b). This indicates the successful substitution of the trivalent Ce^3+^ ions into the perovskite crystal lattice.

The surface chemistry of the as-designed perovskites plays a vital role in their applications in catalysis, especially under harsh environmental conditions. In Table 2, we summarized the results of the BET scan of LaCoO_3_ and their Ce^3+^ substituted perovskites. The specific surface areas pore sizes, and pore volumes were calculated from the respective nitrogen adsorption isotherm. On the textural properties, such as specific surface areas, pore sizes, and pore volumes summarized in Table 2, the as-synthesized LaCoO_3_ has BET surface area of 3.9 m^2^ g^−1^. This slight improvement in the surface area could be due to the formation of La-Ce-Co-O network after the introduction of ceria in the crystal lattice. La_0.95_Ce_0.05_CoO_3_ has BET surface area of 4.7 m^2^ g^−1^. However, upon further addition of cerium ion from 5, to 7, and finally to 10%, the surfaces of the catalysts decreased from 4.4 to 4.3 m^2^ g^−1^.

**Table 2:** Textural properties of the Ce-doped perovskites (La_1-x_Ce_x_CoO_3_).

Nominal composition	Single point BET (m^2^ g^−1^)	Multi point BET (m^2^ g^−1^)	Pore volume (cm^3^ g^−1^)	Pore size (A)
LaCoO_3_	3.6	3.9	0.002	20.37
La_0.95_Ce_0.05_CoO_3_	4.4	4.7	0.0008	18.65
La_0.93_Ce_0.07_CoO_3_	4.05	4.4	0.0007	18.57
La_0.90_Ce_0.10_CoO_3_	4.05	4.3	0.03	303.20

**Figure 3:**
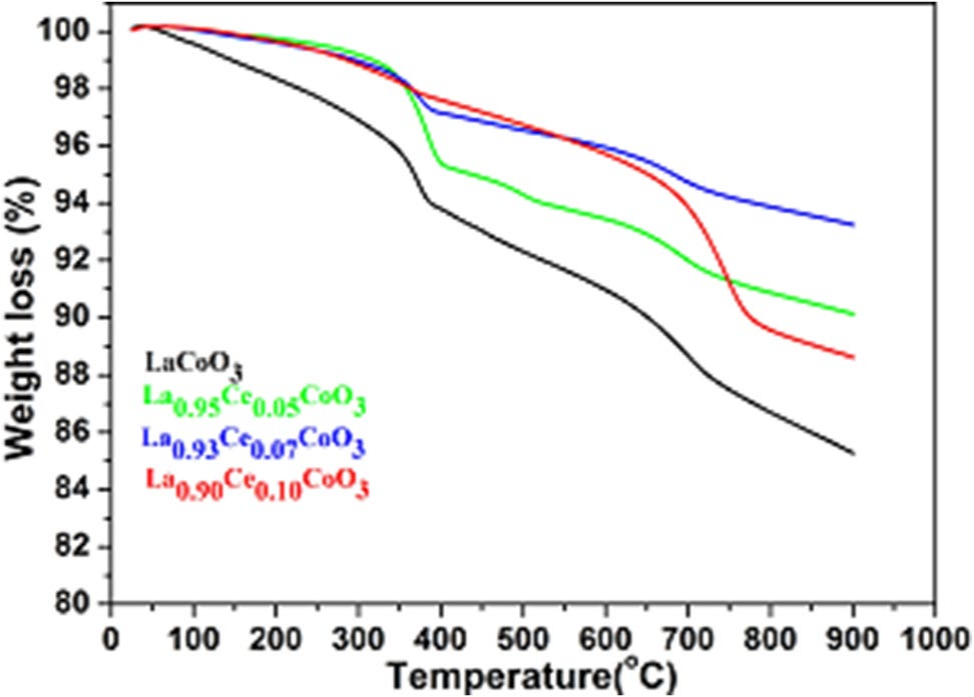
Thermo-gravimetric analysis of LaCoO_3_, La_0.95_Ce_0.05_CoO_3_, La_0.93_Ce_0.07_CoO_3_ and La_0.90_Ce_0.10_CoO_3_ nanoparticles.

The as- synthesized powders were thermally analyzed by TG-DTG measurements. All thermograms shown in Fig. 3 show similarities in shape and illustrated three-step decomposition. In case of LaCoO_3_ perovskite, 6% weight loss was observed in the temperature range from 0 to 395 °C. The initial weight loss is attributed to the removal of surface-adsorbed water molecules and organic moieties. The second weight loss at ~ 6% in the temperature range 395–727 °C is attributed to the removal of crystalline water molecules. A sluggish weight loss of ~ 3% is observed in between 727 and 900 °C temperature, which is assigned to the burning or combustion of lattice oxygen with airborne carbonates to form the perovskite structure. Such observations are in accord with previous literature reports^42–46^. It is worth noting that, in increasing the Ce ion substitution concentration in perovskite lattice, the quantity of weight loss has also increased as seen in the TGA data of La_0.90_Ce_0.10_CoO_3_. We expected this since increasing the doping concentration in perovskite lattice would also increase the lattice distortion within the crystal matrix, because of the reduced bond distance. As verified from the XRD data, increasing doping creates a large number of oxygen vacancies, which decompose or burn at a higher temperature. All thermograms reveal similar thermal decomposition temperatures. Except in the differences in weight losses, this may be due to the surface attached water molecules and organic moieties, which alter the crystallinity of the materials.

**Figure 4:**
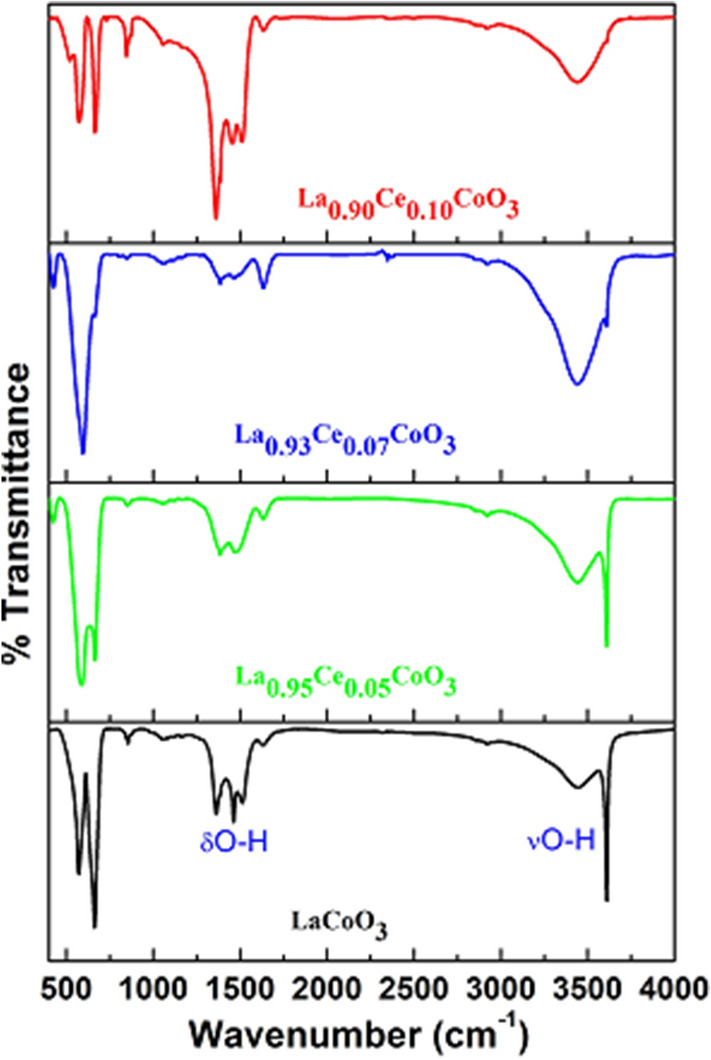
FTIR spectra of LaCoO_3_, La_0.95_Ce_0.05_CoO_3_, La_0.93_Ce_0.07_CoO_3_ and La_0.90_Ce_0.10_CoO_3_ nanoparticle.

FTIR spectra of the as-synthesized perovskites are illustrated in Fig. 4. FTIR spectra of all perovskites exhibit diffused band at around 3,450 cm^−1^ along with two low-intensity infrared absorption bands located at 1,480 and 1,360 cm^−1^, which are assigned to the O–H stretching, bending and scissoring vibrational modes of physically adsorbed H_2_O molecules over the exterior of perovskites^42^. The observed strong intensity infrared band at low frequency (below 700 cm^−1^) is ascribed to the symmetric vibrational modes of La-Ce-Co-O network^47^. Clearly observed from the FTIR spectra of the doped sample, the infrared absorption band at lower frequency are progressively shifted from 572 cm^−1^ to the higher frequency (590 cm^−1^) when increasing the Ce ions doping quantity. Based on the force constant phenomenon, the Ce^3+^ ion (1.12) is more electronegative than their respective La^3+^ ion (1.10), it, therefore, implies that substitution of Ce can shift the La–O bonding towards longer frequency by forming the Ce–O bonds^48^.

**Figure 5:**
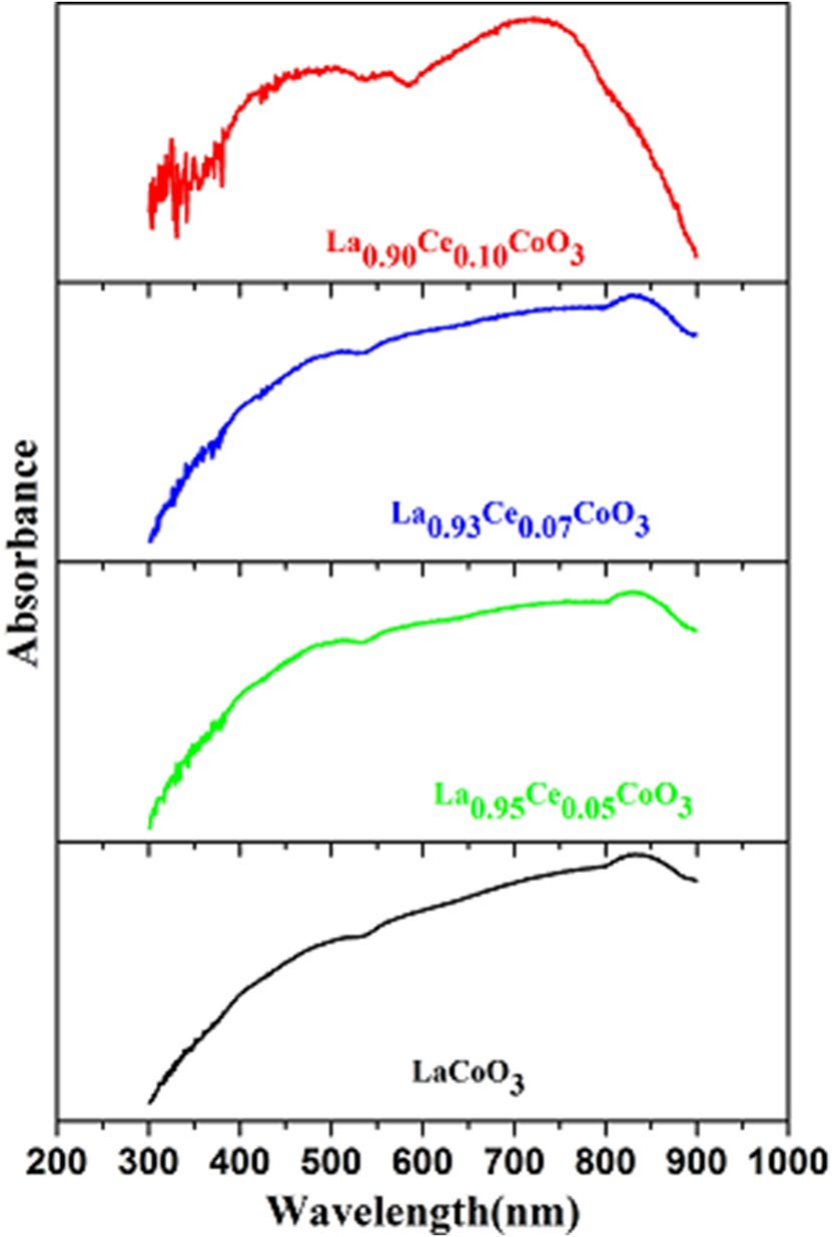
UV–Vis absorption spectra of LaCoO_3_, La_0.95_Ce_0.05_CoO_3_, La_0.93_Ce_0.07_CoO_3_ and La_0.90_Ce_0.10_CoO_3_ nanoparticles.

**Figure 6:**
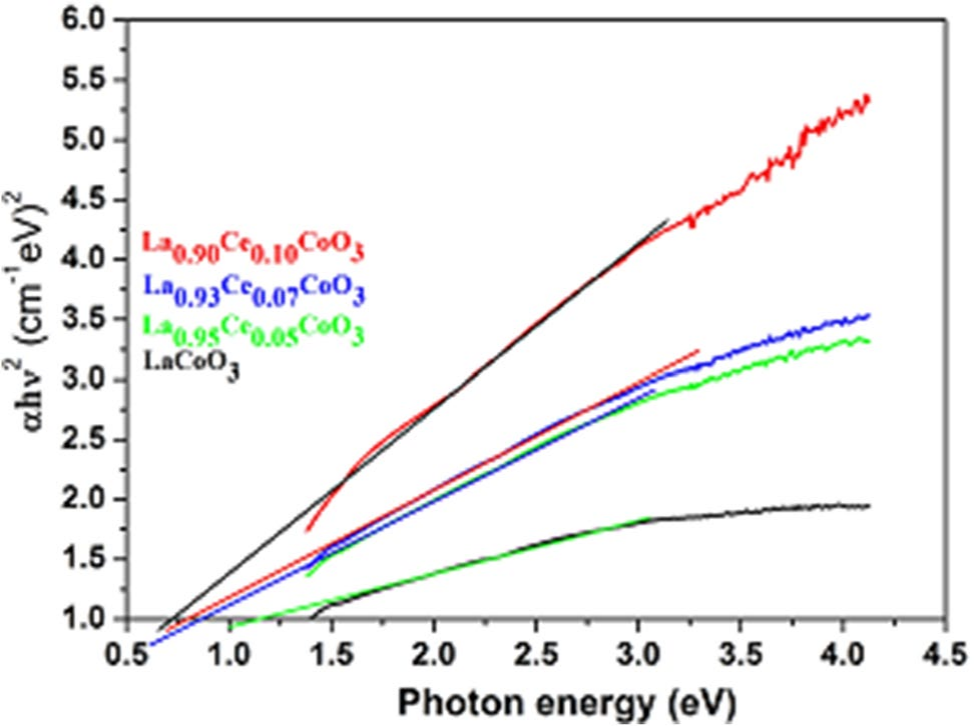
The plot of αhγ^2^ (cm^−1^ eV)^2^ vs. Photon energy (eV) LaCoO_3_, La_0.95_Ce_0.05_CoO_3_, La_0.93_Ce_0.07_CoO_3_ and La_0.90_Ce_0.10_CoO_3_ nanoparticles.

The optical absorption spectra were measured to determine the optical properties, such as the energy band gab (E_g_) of the perovskites materials. It is commonly known that cobalt and ceria ions reveal strong absorption in UV/Vis region. As demonstrated in Fig. 5, all perovskites exhibited broad absorption features in the whole UV/Visible region, even to extended some portion of the near infrared region, because of charge transfer band from O_2_ to 2p orbitals^49^. The E_g_ values are normally estimated by fitting the absorption data into Tauc formula by extrapolating the linear portions of the curves to absorption to zero^22^. The experimentally calculated E_g_ values of all samples are found to be 1.13, 0.86, 0.79 and 0.71 eV for LaCoO_3_, La_0.95_Ce0.05CoO_3_, La_0.93_Ce_0.07_CoO_3_ and La_0.90_Ce_0.10_CoO_3_, respectively, as shown in Fig. 6. It is observed from the figure that the E_g_ values has gradually reduced by when increasing the amount of the doping Ce ions. It is known that the valence band of LaCoO_3_ consists of O 2p charge transfer band, whereas the conduction band is made up of Ce (4f → 5d) orbitals^49^. The hybridization, therefore, occurs between Ce 5d and oxygen (2p) orbitals due to the similarity in their energy and spatial overlaps. Additionally, the enhancement in the electronegativity of the Ce metal increases the magnitude of metal–oxygen hybridization, resulting into a shift of the metal 5d orbitals and oxygen 2p orbital which are closer in energies^48^. It is also known that Ce has high electronegativity than that of La, so that in the substitution of Ce ion quantity in LaCoO_3_, an additional energy level below the conduction band is created and thus lowers the E_g_ values as well. Moreover, the E_g_ values gradually decrease when increasing the Ce ion quantity, which indicates that the number of photons absorbed by the catalysts increases with a decrease in the band gap. This subsequently increases the electron and hole density in the conduction band and valence band, respectively^50^.

### Texture, redox properties and chemical states

**Figure 7:**
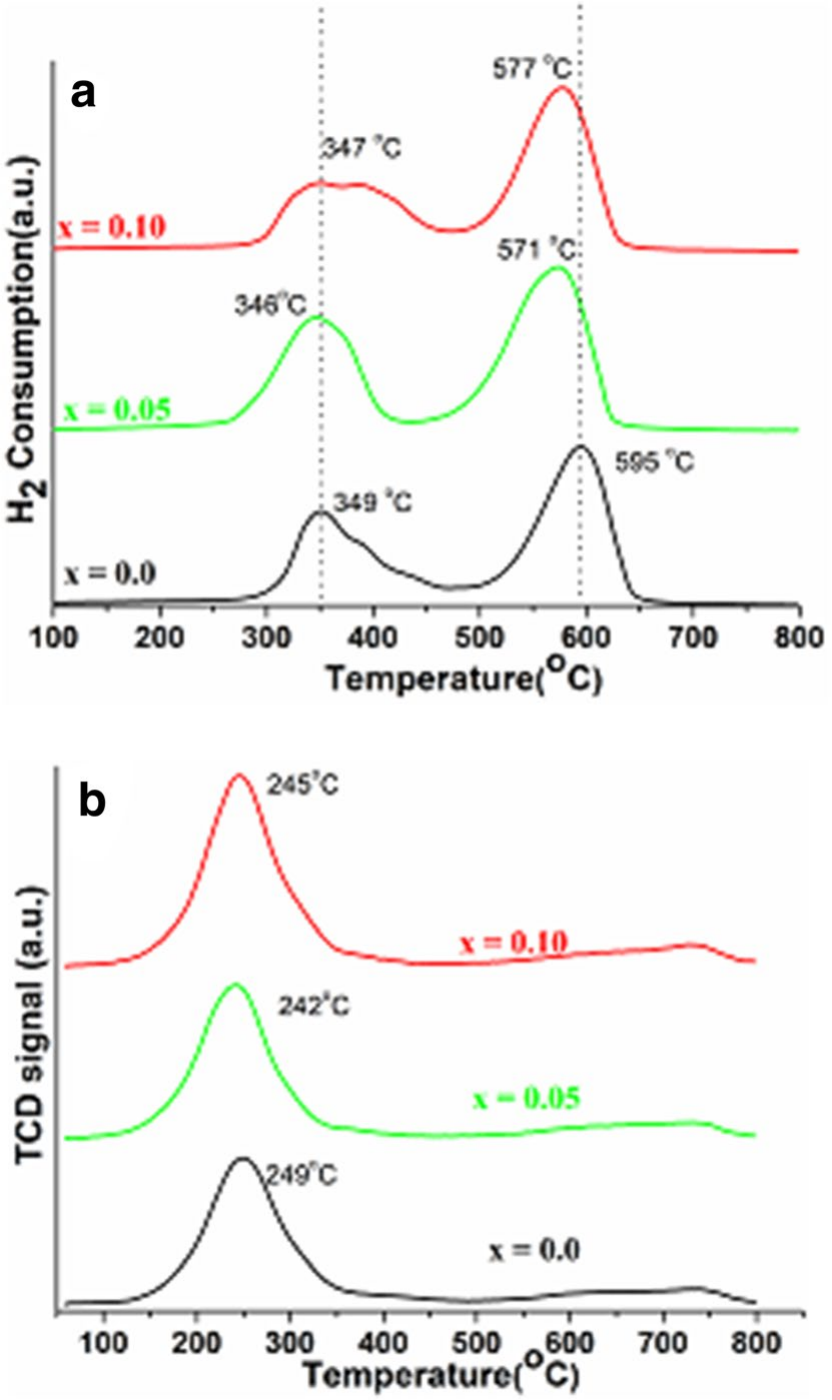
(**a**) TPR and (**b**) TPO profile of LaCoO_3_, La_0.95_Ce_0.05_CoO_3_ and La_0.90_Ce_0.10_CoO_3_ perovskite nanoparticles.

Figure 7a,b show the redox behavior of the fabricated catalysts. Figure 7a illustrates the reduction profiles of LaCoO_3_ and their Ce doped perovskite materials. Two reduction peaks can be identified for all samples, indicating a stepwise reduction of perovskites and their derivatives. An observed low-temperature peak in between 260 and 460 °C, whereas high- temperature peaks were observed between 475 and 650 °C temperature ranges. At lower temperature H_2_-consumption peak is attributed to the reduction of Co^3+^ to Co^2+^ and the reduction of the excess O_2_ species and the chemisorbed oxygen on the surface of the catalysts, while reduction peak observed at high temperature (595 °C), is ascribed to the reduction of Co^2+^ into metallic cobalt (Co^0^), and the reduction of chemisorbed oxygen caused by lattice defects, as reported in literature^34,51–54^. With the introduction of 5% Ce, the H_2_-consumption peaks progressively shifted towards lower temperature. This could be due to the insertion of Ce ion, which significantly accelerate the reduction of lattice oxygen species, thus resulting to the weakening of Co–O bond^21,34,55–57^. As shown in Fig. 7a, increasing the ceria content from 5 to 10%, shifts both the reduction peaks shifted towards higher temperatures. The shifting of reduction peaks at higher temperature, when increasing the cerium content, may be caused by the existence of Ce ions in the framework, which provides electrons to hydrogen and thus delay the reduction of Co species, in accordance with the literature^31,34,57^. Additionally, it indicates that incorporation of large amount of Ce ions into the LaCoO_3_ crystal lattice enhances the reduction of Co species^34^. Furthermore, it also suggests that substitution of Ce ions into the LaCoO_3_ crystal lattice could have promoted the formation of structural defects.

Temperature program oxidation study was performed to explore the catalytic oxidation and the oxygen species adsorbed on the surface of catalysts. As shown in Fig. 7b, the reverse transformations of Co oxidation states are observed by the TPO analysis. It shows an observed peak at low temperature in between 110 and 365 °C, typically assigned to the transition of metallic Co^0^ to Co^2+^ , while the peak in between 623 and 790 °C shows the oxidation from Co^2+^ to Co^3+^. These results are in good agreement with reported literature^58–60^.

**Figure 8:**
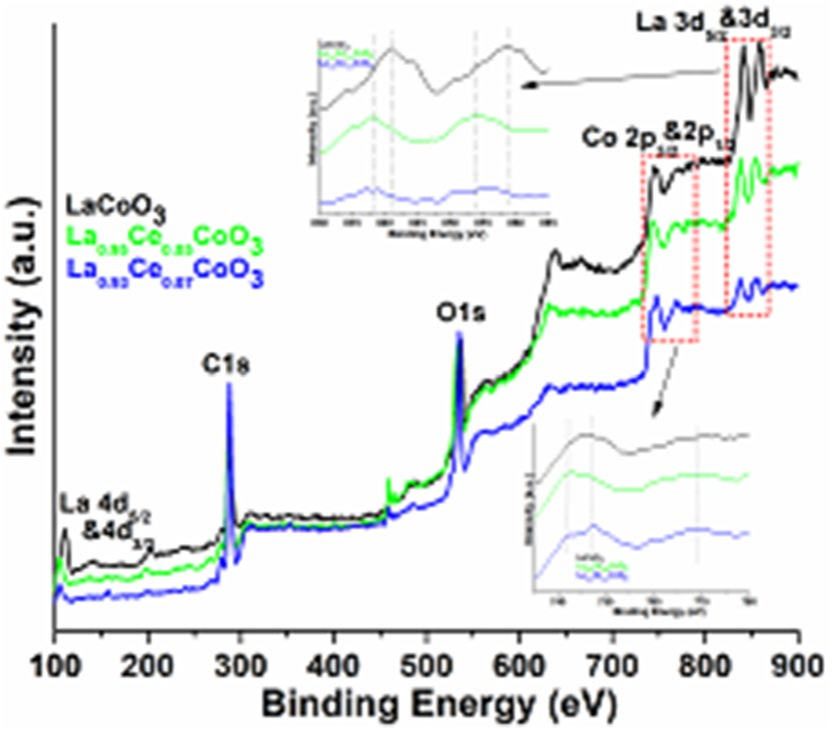
XPS analysis of the La 3d_3/2&5/2_ spectra recorded for the LaCoO_3_, La_0.95_Ce_0.05_CoO_3_, and La_0.93_Ce_0.07_CoO_3_ nanoparticles.

XPS analysis was performed to determine the valence states of the elements in the as-prepared perovskites. The wide spectra of LaCoO_3_, 5, 7 and 10% doped Ce ions are shown in Fig. 8. The results reveal the existence of La (3d), Co (2p), C (1s) and O (1s) atoms in perovskites. A strong peak below 300 eV was observed for the adventitious carbon on the surface materials^49,61^. Two strong intensity peaks at 840.9 and 858.6 eV are assigned the spin–orbit splitting of 3d_5/2_ and 3d_3/2_ of the La(III) ions in all perovskites^61,62^. The XPS spectrum of LaCoO_3_ illustrates three prominent peaks along with shake-up satellites at the binding energies of (780, 784.2), (789.9, 792), and (795, 799 eV) and ascribed to Co 2*p*_*3/2*_, Co 2*p*_*1/2*_, respectively. This indicates that Co ion in LaCoO_3_ is mainly in the trivalent state^63–65^. The occurrence of shake-up satellite peaks at 784.2, 789.9, 792, and 799 eV verifies the characteristic peaks of Co^2+^ ion. These results are well consistent with the previous literature reports^39,65^. Figure 8 has clearly revealed the influence of ceria doping, that is, in the insertion of ceria in perovskite lattice, the binding energies are progressively shifted towards the lower sides.

**Figure 9:**
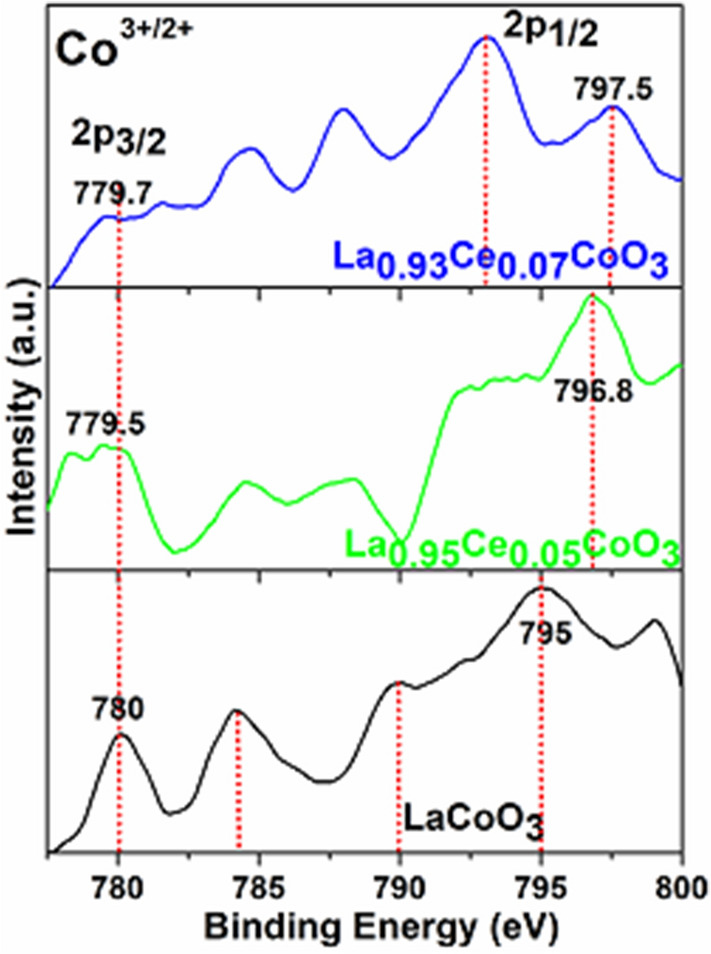
XPS analysis of the Co 2p spectra recorded for the LaCoO_3_, La_0.95_Ce_0.05_CoO_3_, and La_0.93_Ce_0.07_CoO_3_ nanoparticles.

Figure 9 demonstrates the narrow-range Co 2p XPS spectra of the LaCoO_3_ and other perovskites. It is observed that the binding energy of Co 2p_3/2_ in La_0.95_Ce_0.05_CoO_3_ perovskite is shifted to lower binding energy at 778, with respect to the LaCoO_3_ sample. It could be due to some amounts of Co^3+^ ions being transformed into Co^4+^ state, after the incorporation of Ce ions in the perovskite lattice. We believe Ce ion is in the tetravalent state, because of its high stability, then in their trivalent state. Furthermore, to maintain the electronic balance, after the replacement of trivalent La by higher valence Ce^4+^, trivalent Co is bound to transform into a higher valence^66^. Similarly, in the La_0.93_Ce_0.07_CoO_3_ perovskite, peaks are shifted to lower side, implying that substitution of Ce ion induces some amount of Co^3+^ to transform into Co^4+^ in the samples, which is analogous to the literature reported for Co_2_O_3_^65,66^. It, therefore, verifies that the main valence of Co ions is, indeed, trivalent^67^.

**Figure 10:**
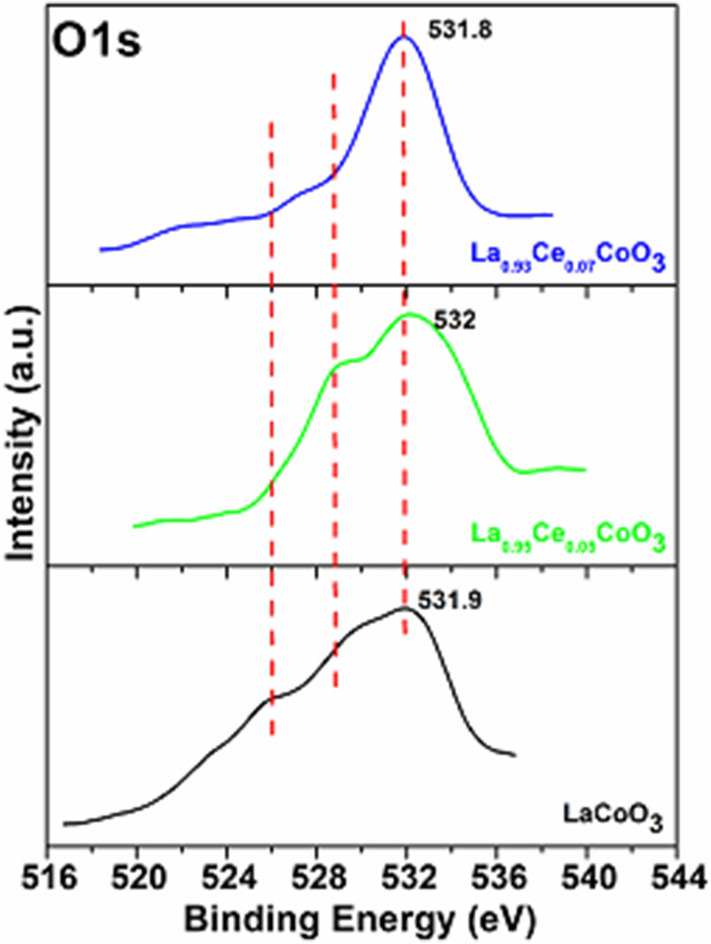
XPS analysis of the O1s spectra recorded for the LaCoO_3_, La_0.95_Ce_0.05_CoO_3_, and La_0.93_Ce_0.07_CoO_3_ nanoparticles.

Figure 10 demonstrates the narrow range O1s XPS spectra of the LaCoO_3_ and other perovskites. XPS spectra of O1s reveal two dominant components of different types of oxygen species. The XPS spectra of LaCoO_3_ exhibited a low rising peak between 523 and 529 eV, which is attributed to the lattice oxygen molecules. Another broad peak intensity at 531.9 eV indicates the surface adsorbed oxygen species. Noticeably, the surface adsorbed oxygen species exhibits a higher mobility, than their respective surface lattice oxygen. The comparative content of adsorbed oxygen vacancies is usually considered as a parameter to evaluate the catalytic performance of the catalyst. In the comparative analysis, in adding Ce ion impurities into the La_0.95_Ce_0.05_CoO_3_ perovskite lattice, the relative contents of adsorbed oxygen vacancies remarkably rise and shift towards the higher binding energy. However, by further increasing the Ce ion concentration in La_0.93_Ce_0.07_CoO_3_ perovskite lattice, the contents of adsorbed oxygen vacancies are shown to be lower than that of La_0.95_Ce_0.05_CoO_3_ perovskite, which indicates that the La_0.95_Ce_0.05_CoO_3_ perovskite possesses higher oxygen adsorbed vacancies than the La_0.93_Ce_0.07_CoO_3_ perovskite. A significant decrease in the area of oxygen adsorbed species when on the addition of a higher quantity of Ce ions was observed, which indicates that the excessive introduction of Ce ions would compensate charge with Co ions, resulting in a reduction on oxygen species^67^. Additionally, the La_0.95_Ce_0.05_CoO_3_ perovskite provides a larger specific surface area, which can significantly enhance the adsorption capability of oxygen. As a result, the La_0.95_Ce_0.05_CoO_3_ perovskite possess higher catalytic activity due to the high surface area and high mobility of the oxygen adsorbed vacancies. This will be further discussed below.

### Aerobic oxidation of benzyl alcohol

**Figure 11:**
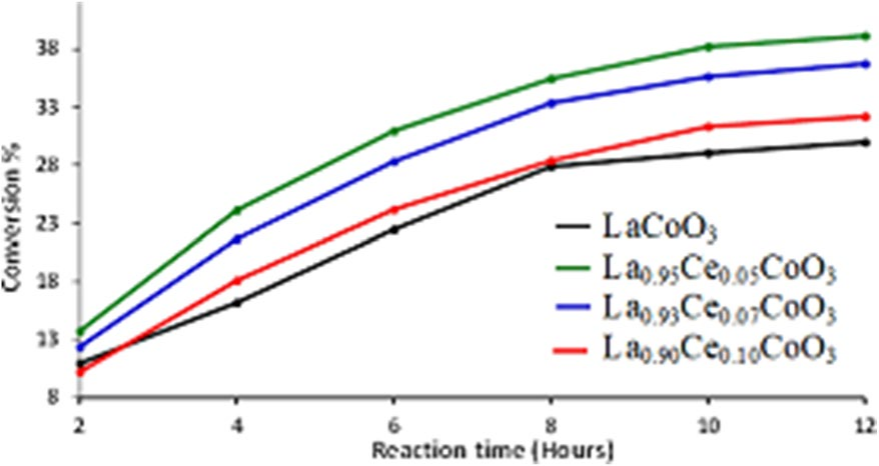
Graphical representation of the kinetics of oxidation of benzyl alcohol to benzaldehyde employing perovskites.

The prepared catalysts were tested for oxidation of benzyl alcohol to benzaldehyde. The results are shown in Table 3. Benzaldehyde is the main product, with a negligible amount of benzoic acid as a by-product. It was found that the un-doped perovskite, i.e., LaCoO_3_ starts off with a benzyl alcohol conversion of 11% within 2 h. However when the reaction was continued for 12 h, and it yielded 30% conversion of benzyl alcohol with the specific activity of 0.25 mmol g^−1^ h^−1^. It is also interesting to make a further comparison of the three catalysts with different cerium contents. Figure 11 shows that insertion of Ce into LaCoO_3_ perovskite lattice remarkably improves the catalytic activities, and maintains the high selectivity towards benzaldehyde. On substitution of 5% Ce ions into LaCoO_3_ perovskites a significant enhancement in catalytic performance was observed such as catalyst yielded a 40% conversion product with the specific activity of 0.33 mmol g^−1^ h^−1^. It is worth noting that the addition of cerium ions into the perovskite lattice influence the surface vacancies, which significantly enhanced the synergistic effect between the Ce^3+/4+^ and Co^2+/3+^ ions. It is well-known fact that Ce^3+/4+^ exhibited reversible oxidation states, resulting cerium ions significantly improve the catalytic activity of the catalyst. It is also evident from XPS analysis Co existed in trivalent state and generally Ce exist in tetravalent state, so that to keep the charge equilibrium in perovskite oxide, Ce should promote the excessive oxide anion on the surface and, thereby, oxygen is easily delivered from the surface for CO oxidation. Although, XPS analysis illustrate enrichment of Co at the surface, which stimulates the oxygen storage capacity. However, when the percentage composition of Ce is further increased in the perovskite system, the catalytic performance decreased. It could be due to the low quantity of Ce^3+/4+^ions accommodate within the lattice and enhanced the active sites, whereas high quantity doping surplus within the lattice and suppressed the active sites of the catalyst. Similar trend is observed in the case of the surface area, which decreases from 4.732 to 4.271 m^2^ g^−1^. This indicates that the Ce atom in the perovskite could have hindered the active site of the perovskite leading to depreciation of catalytic performance and of the surface area of the catalysts. These observed results indicated that doping of ceria ions played a crucial role in the improvement of catalytic activity, because of cerium ions induces high oxygen ion mobility, resulting it increase the redox characteristics of the perovskites as verified from XPS analysis.

**Table 3:** Effect on the catalytic properties with the incorporation of Ce in the perovskite system La_1-x_Ce_x_CoO_3_.

Catalyst	Conv. (%)	Sel. (%)	Specific activity (mmol g^−1^ h^−1^)	TON	TOF (h^−1^)
LaCoO_3_	29.97	> 99	0.25	–	–
La_0.95_Ce_0.05_CoO_3_	39.14	> 99	0.33	1,096.82	91.40
La_0.93_Ce_0.07_CoO_3_	36.70	> 99	0.31	735.59	61.30
La_0.9_Ce_0.1_CoO_3_	32.18	> 99	0.27	450.89	37.57

Reaction conditions: 2 mmol of benzyl alcohol, calcination temperature at 300 ºC, oxygen with rate 20 mL min^−1^, 0.3 g of catalyst, 10 mL of toluene, reaction temperature at 100 ºC, and 12 h of reaction time.

**Figure 12:**
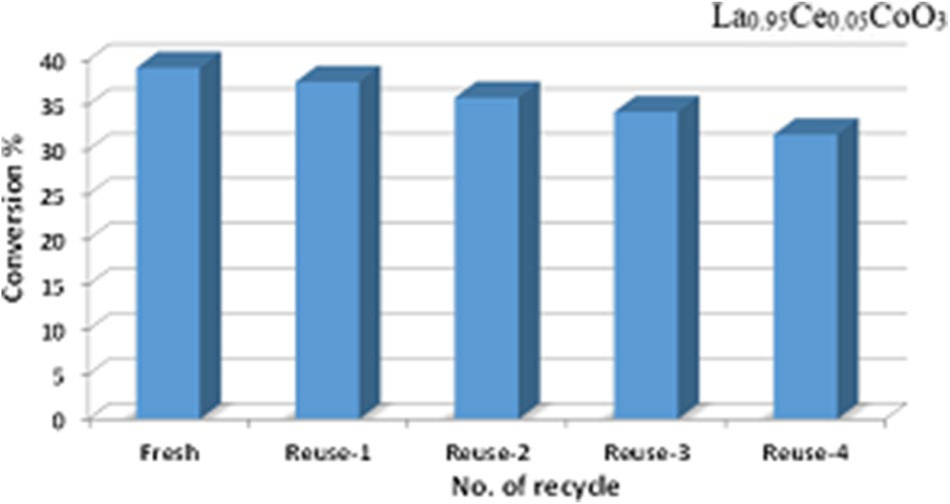
Recyclability of La_0.95_Co_0.05_CeO_3_ for the aerial oxidation of benzyl alcohol. (Reaction conditions: 2 mmol of benzyl alcohol, calcination temperature at 300 ºC, oxygen with rate 20 mL min^−1^, 0.3 g of catalyst, 10 mL of toluene, reaction temperature at 100 °C, and 12 h of reaction time).

The catalyst recovery has significant importance both from the industrial and academic point of view. In this work, the recyclability of La_0.95_Ce_0.05_CoO_3_ for the oxidation of benzyl alcohol via molecular O_2_ was also studied under optimized conditions. Upon addition of fresh toluene, the mixture was filtered to recover the catalyst by a simple filtration process. The filtered catalyst was washed successively with toluene and dried at 100 °C for 12 h (Fig. 12). The catalyst was used for several times and was found that the catalytic performance has depreciated by 1.55% in the first reuse. However further reuse leads to further loss in catalytic activity And upon 4 times of reuse, the catalyst yielded a 31.7% conversion of benzyl alcohol after 12 h of reaction time, which is 7.44% less than the first time use of the catalyst, indicating that the catalyst is marginally stable for re-use, and can be further modified to increase its catalytic performance and reusability.

## Conclusions

A series of cerium doped LaCoO_3_ perovskites were successfully prepared by sol–gel chemical process. In the comparative results, the substitution of Ce ions in perovskite lattice has remarkably improved the crystallinity, thermal, optical, surface properties and redox properties of the perovskites. The TPR/TPO study has verified that the low concentration doping of Ce ion enhances the reducing ability of the LaCoO_3_ perovskite at low temperature. When the resulting catalysts were tested in the liquid-phase aerobic oxidation of benzyl alcohol to benzaldehyde using molecular O_2_ as a green oxidant, the catalytic activity tests show that the activity of catalyst depends strongly on the percentage doping of cerium. In these newly designed perovskites, La_0.95_Ce_0.05_CoO_3_, the perovskite has demonstrated an excellent catalytic activity towards benzyl alcohol oxidation with high selectivity The catalyst can be recycled several times without any loss in conversion and selectivity. This, therefore, suggests its reusability and stability. Easy product recovery and recycling efficiency along with high selectivity of this material could be useful for the synthesis of different chemicals under eco-friendly conditions.
